# Sub-diffraction Limit Localization of Proteins in Volumetric Space Using Bayesian Restoration of Fluorescence Images from Ultrathin Specimens

**DOI:** 10.1371/journal.pcbi.1002671

**Published:** 2012-08-30

**Authors:** Gordon Wang, Stephen J. Smith

**Affiliations:** Department of Molecular and Cellular Physiology, Beckman Center, Stanford University, Palo Alto, California, United States of America; Indiana University, United States of America

## Abstract

Photon diffraction limits the resolution of conventional light microscopy at the lateral focal plane to 0.61λ/NA (λ = wavelength of light, NA = numerical aperture of the objective) and at the axial plane to 1.4nλ/NA^2^ (n = refractive index of the imaging medium, 1.51 for oil immersion), which with visible wavelengths and a 1.4NA oil immersion objective is ∼220 nm and ∼600 nm in the lateral plane and axial plane respectively. This volumetric resolution is too large for the proper localization of protein clustering in subcellular structures. Here we combine the newly developed proteomic imaging technique, Array Tomography (AT), with its native 50–100 nm axial resolution achieved by physical sectioning of resin embedded tissue, and a 2D maximum likelihood deconvolution method, based on Bayes' rule, which significantly improves the resolution of protein puncta in the lateral plane to allow accurate and fast computational segmentation and analysis of labeled proteins. The physical sectioning of AT allows tissue specimens to be imaged at the physical optimum of modern high NA plan-apochormatic objectives. This translates to images that have little out of focus light, minimal aberrations and wave-front distortions. Thus, AT is able to provide images with truly invariant point spread functions (PSF), a property critical for accurate deconvolution. We show that AT with deconvolution increases the volumetric analytical fidelity of protein localization by significantly improving the modulation of high spatial frequencies up to and potentially beyond the spatial frequency cut-off of the objective. Moreover, we are able to achieve this improvement with no noticeable introduction of noise or artifacts and arrive at object segmentation and localization accuracies on par with image volumes captured using commercial implementations of super-resolution microscopes.

## Author Summary

Biological function at its fundamental level involves molecular interactions on a nanometer scale, and it is this reason that biological imaging has pushed for increasingly better resolution. Light microscopy is highly prevalent in biology due to its combination of large field of view, simple sample preparation, cost effective usage and relatively high tolerance by biological samples. The problem with light microscopy is that diffraction of light limits the resolution of achievable images to hundreds of nanometers in volumetric space, which is much too low for the accurate localization of proteins in subcellular organelle or structures, such as the synapse of a neuron. Super-resolution light microscopy is now available, but its implementation usually requires technically complex and expensive imaging systems. In this paper, we demonstrate a method that combines physical thin sectioning of tissue with Bayesian based deconvolution of conventional, fluorescent microscopy to achieve volumetric resolution well below the diffraction limit, and that using this method we are able to greatly improve the computational segmentation and localization of labeled proteins in a reconstructed volume of brain tissue.

## Introduction

The spatial resolution and definition of the cellular protein matrix is fundamental to the characterization and analysis of cellular function. The accurate resolution of sub-organelle protein localization, in tissue, on a proteomic scale is immensely useful. It is with this in mind that we developed Array Tomography (AT), a proteomic imaging technique. AT uses ribbon arrays of ultrathin (50–100 nm) physical sections of resin-embedded, fixed tissue for multiple rounds of immunohistological detection, which produces a rich, high-dimensional matrix of protein information in an ex-vivo context [1], [2]. AT allows the collection of 30+ channels of protein information in a cubic millimeter volume of brain tissue [1], [2]. This information is only useful if we can, with spatial accuracy, localize spatially aggregated protein units within cellular structures and in relation to all other imaged protein channels. This places a premium on the computational segmentation of objects in the image volume, and is highly dependent on resolution and contrast.

The axial resolution of AT image volumes is limited only by the physical sectioning, which is 50–100 nm and is far smaller than the diffraction limited axial resolution of most microsocopes (∼385 nm). However, the lateral resolution of AT image volumes is still limited by the Abbe diffraction limit (∼200 nm for visible wavelengths) [3], [4]. At that lateral resolution, the segmentation of densely packed proteins, such as Synapsin (a highly abundant presynaptic protein in the brain), is unreliable and difficult. Recently, AT was combined with direct stochastical optical reconstruction microscopy (dSTORM) to achieve lateral resolution of ∼40 nm [5]. However, dSTORM imaging is time consuming and requires specialized microscopes. Thus, we investigated deconvolution as a simple and efficient method to improve our resolution in AT. The reason for considering deconvolution is that the physical sectioning of AT provides full removal of out of focus light, and the ideal correction of refractive index, astigmatism, coma, spherical aberration and curvature of field [1]. Moreover, the thinness of the tissue coupled with the direct placement of the sample onto glass also means that the heterogeneity of refractive indexes in normal biological samples is not present, which further eliminates sources of aberration and wave-front distortions. These properties, which are not present in most imaging techniques, allow AT to produces image volumes where the point spread function (PSF) is truly spatially invariant throughout, which makes these images an ideal substrate for deconvolution.

Deconvolution is a method by which the diffracted light is computationally returned back into its actual source using either an idealized or empirically measured PSF [6], [3], [4]. The PSF describes the diffraction of light from a point source. Specimens in the image are blurred by the PSF at a point by point basis. This blurring can be considered a convolution operation on the image [7], [8], [3], if it is linear (each point source in the image sums their intensity linearly) and shift invariant (the PSF is the same for the entire field of view). Wide-field is such an imaging systems [8], although in actual biological tissue the heterogeneity and depth of the tissue volume does introduce aberrations, wave-front distortions and out of focus light contributions that can cause significant deviations in the PSF across the image volume, which adversely affect the quality of deconvolution. This is not the case for AT thin sections where the PSFs are truly spatially invariant. Moreover, it might be easier to appreciate the advantages of thin physical sections by thinking about the analogy to conventional optical sectioning microscopes such as confocals. Confocals achieve optical sectioning by using a pinhole to reject out of focus light. This improves image quality by increasing the collection of high spatial frequency information in the image, but this comes at a cost of reduced signal to noise, due to the rejection of in focus light by the pinhole. AT physically removes all out of focus light sources, which means that AT does not need to use a pinhole for optical sectioning thus allowing it to provide both high signal to noise (which, in normal confocal microscopy, would be maximized by a large-diameter pinhole) and measurement of high-frequency spatial information (which would be maximized by a small-diameter pinhole) [9], [3].

The content of high-frequency information in the image is reflected in the bandwidth of the Optical Transfer Function (OTF), which is the Fourier Transform (FT) of the PSF. In confocal the OTF bandwidth varies inversely with pinhole diameter [9], [3]. The OTF determines the actual spatial frequencies transferred to the recorded image. Thus, if the OTF were small at high spatial frequencies (as is the case for an expanded confocal pinhole or a conventional wide-field setup), the high-frequency components of the specimen would be greatly attenuated, causing blurring and decreased resolution. Interestingly, the OTF of a theoretical infinitely-small pinhole would have twice the bandwidth of a standard wide-field OTF [10], [9]. In AT, we approximate this ideal pinhole with physical sectioning, and combined with the spatially invariant PSF, allow us to perform deconvolution at its mathematical optimum, which should, with the correct algorithm, allow us to greatly increase the magnitude of recovery for high spatial frequency information in the OTF up to the physical bandwidth limit, which is defined by diffraction.

Richardson-Lucy deconvolution (RL) is a Bayesian based expectation maximizing deconvolution method originally developed for the restoration of images in astronomy [11]–[14]. RL has several advantages for AT images. It assumes the non-negativity of the observations and that the statistic of the associated noise follows a Poisson distribution, which is appropriate for fluorescent images [15], [13], [16]. RL is globally and locally intensity-conserving at each iteration [11], [12], thus ensuring that intensity data remain quantifiable after deconvolution [13], [15]. RL is computationally efficient, and the restored images are robust against small errors in the image and the point-spread function (PSF) [12], [11], [17], [15], which makes its real world implementation realistic. Finally, in our tests on AT images, RL significantly out performs other non-Bayesian based deconvolution methods, and has demonstrated a greater than 8 fold increase in the magnitude of spatial frequency recovery up to the diffraction limit, without any measurable introduction of artifact or noise into the images. Moreover, RL in our application demonstrated mathematically a potential for the recovery of spatial frequencies beyond the diffraction limit, which likely contributes to the analytical improvements seen in the analysis of the deconvolved tissue volumes.

Thus, the confluence, in AT, of an essentially two-dimensional sample imaged at the optical optimum of the imaging system (e.g., minimal spherical aberration, optimal refractive index correction, ideal flatness of field, high signal to noise and a spatially invariant PSF) [1], [2] allows AT in combination with RL to achieve volumetric resolution significantly better than the diffraction limit. Using this technique, we demonstrate accurate and clean computational separation of objects in densely labeled tissue volumes.

## Results

### Array Tomography with Deconvolution (ATD)

**Figure 1:**
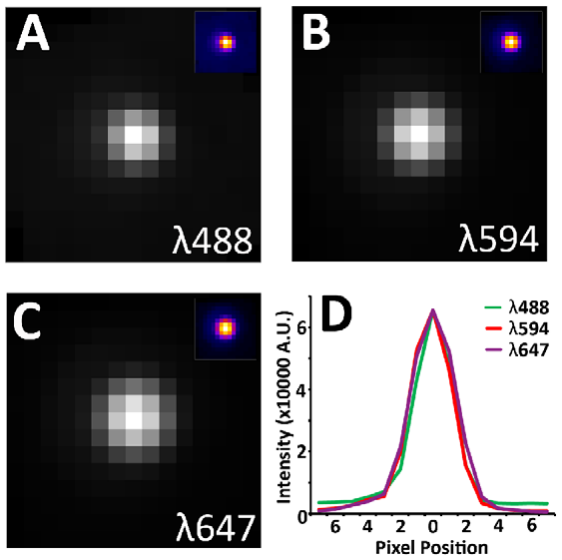
Empirically measure point spread functions (PSF). 110 nm Tetraspeck beads (Invitrogen) are suspended in ethanol. The solution is then applied to ultra-thin (70 nm) tissue arrays and let dry. The arrays are then mounted in mounting medium and imaged. Special care is made to ensure that no more than a single pixel per bead is saturated. Beads from multiple images across the entire field is registered and averaged to produce a single PSF. (A) PSF at 488 nm. Average of 268 beads. (B) PSF at 594 nm. Average of 282 beads. (C) PSF at 647 nm. Average of 335 beads. (d) Cross-sectional plot of each PSF. Note that the width of the PSF increases slightly with increased wavelength.

**Figure 2:**
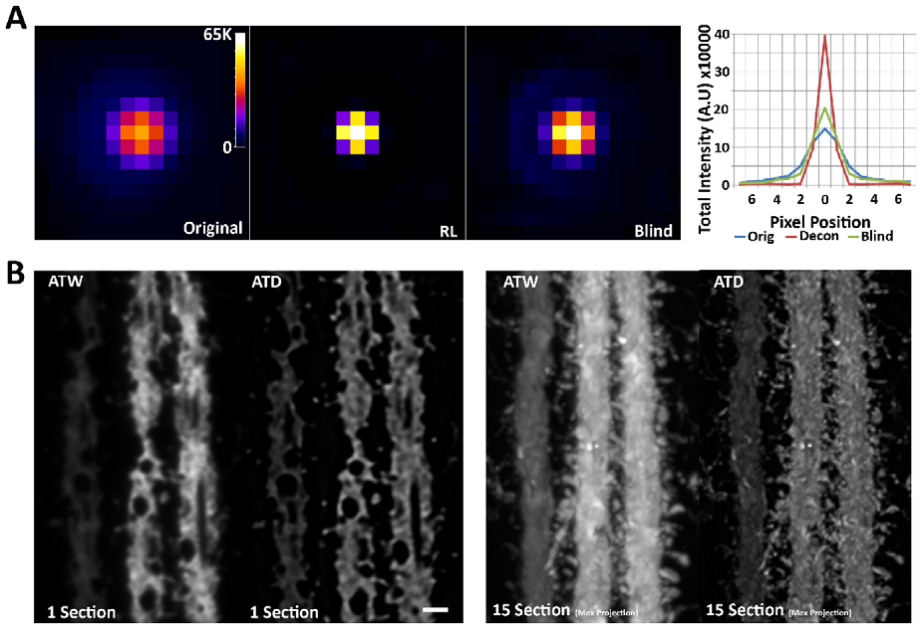
Two-dimensional Richardson-Lucy deconvolution of array tomographic images. (A) Comparison of RL deconvolution with empirical PSF versus blind deconvolution using an initial Gaussian PSF on a single sub-diffraction bead. Both methods are intensity conserving, while RL using an empirical PSF does a much better job of returning the light back to the central pixel. The graph is a plot of the sum of intensities across each column of pixels in the images. Note the conservation of intensity and the dramatic increase in central intensity in the RL plot. (B) Single section and Max projection of AT reconstructed YFP dendrite before and after two-dimensional deconvolution. Spines are clearly resolved in the deconvolved image. Scale bar = 1 um.

Two-dimensional RL deconvolution is used to improve the resolution of protein structures. Initial deconvolution trials using ultra-thin sections seeded with 110 nm beads using RL with a high-quality, low-noise empirical PSF (Figure 1) or blind deconvolution using a hypothetical Gaussian as an initial PSF (Figure 2A) demonstrated that RL performed significantly better, returning most of the diffracted light back into the central pixel (1 pixel = ∼100 nm, 1.4NA Oil objective). Further tests using RL on volumes of YFP labeled dendrites of Layer 5 pyramidal neurons, imaged in traditional wide-field AT (ATW), demonstrated significant improvements in contrast and the visible recovery of high spatial frequency information in the image, which lead to a dramatic qualitative improvement in image quality (Figure 2B).

**Figure 3:**
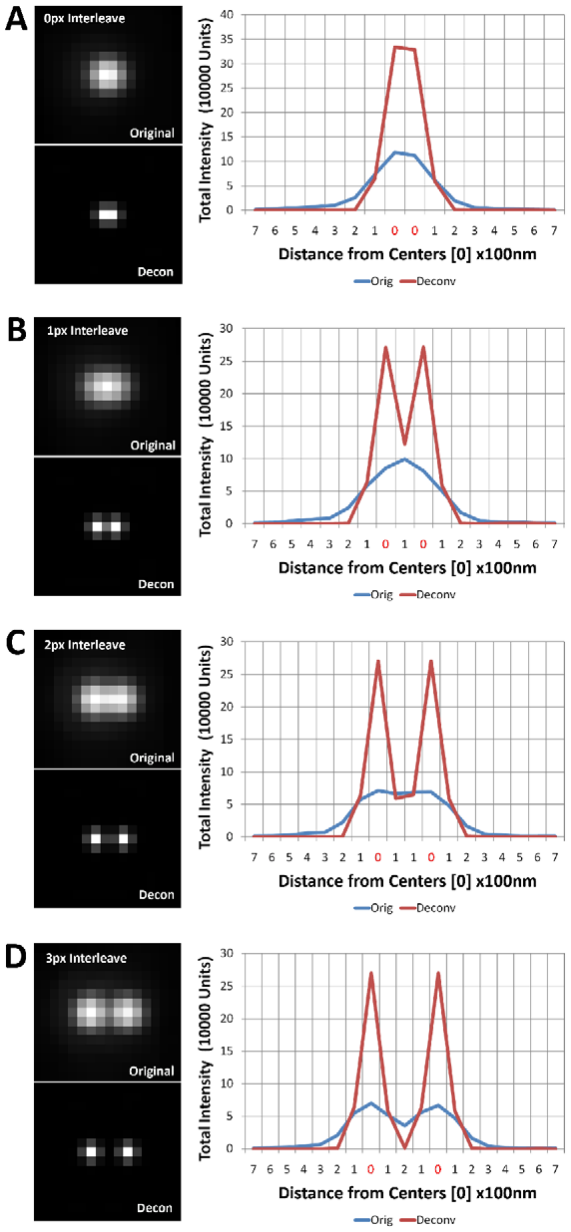
Deconvolution improves the resolvability of adjacent point sources. Here we plot the central cross-sectional profiles of an empirically measured point source, duplicated to simulate two adjoining point sources. Through the linear addition of intensity profiles after the two point sources were shifted in space, we demonstrate the improved resolution of the point sources after deconvolution. Note: 0 along the x axis denotes the center of the two point sources. (A) The centers are shifted by 1 pixel (100 nm) apart, and predictability one cannot resolve the two points in either case, because the centers occupy adjacent pixels. (B) The centers are shifted by 2 pixels (200 nm) apart, and now clearly the deconvolved point sources are resolvable. (C) The centers are shifted by 3 pixels (300 nm) apart. The situation is not different from the 2pixel shift. (D) The centers are shifted by 4 pixels (400 nm), and finally, the non-deconvolved point sources are resolvable. Thus, deconvolution decreases the threshold of resolvability for 4 pixel shift to 2 pixel shift.

This qualitative increase in image quality accompanies a quantitative increase in object separation that can be further demonstrated through a simulation of improved point source discrimination by deconvolution of two adjoining points of light (Figure 3A–D). Within a fluorescent image measured intensity from point sources of light sum linearly [3], [8]. In figure 3 and Figure S1, two point sources are progressively moved further apart, and it is clear in both the image and the cross-sectional plot that after deconvolution the two point sources start to become visibly separate with only a single pixel between them (Figure 3B, S1B), while in the original image the two points only become noticeably separate with 3 pixels between them (Figure 3D, S1D). This demonstrates a theoretical improvement in resolution that pushes the resolvability of point sources in the image to 1pixel separation or 100 nm in our setup.

**Figure 4:**
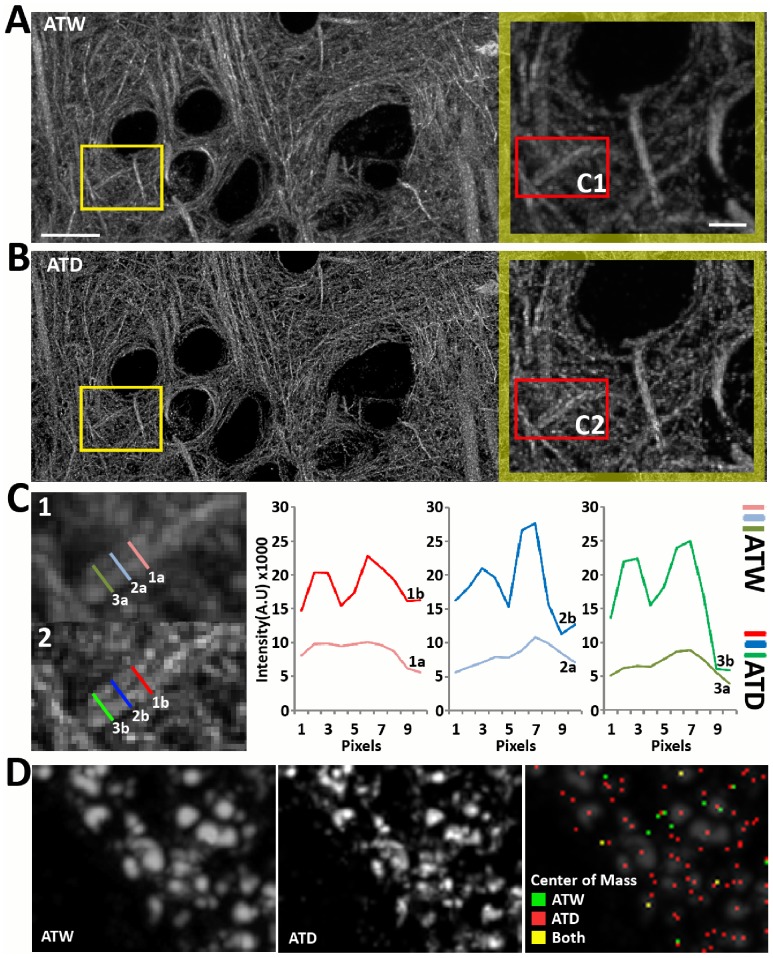
Deconvolution improves computational resolution of fine cellular structures. (A–C) Comparison of microtubules before and after deconvolution. Images are max projection of AT volumes composed of twenty 70 nm sections. (A–B) It is clear that there is more contrast and higher frequency information is more visible in the deconvolved image. Scale Bar 10 um. Blow-up: Scale Bar 2 um. (B–C) We quantify two parallel microtubules separated by one pixel distance at the cross sections marked in the blow-up images in (B). Scale Bar = 1 um. Intensity cross sections (C) along the length of the microtubules show that the peaks of the microtubules are clearly resolved in the deconvolved case, as compared to the original image, where the peaks are barely separated. (D) Deconvolution of Synapsin puncta, a presynaptic protein, makes individual puncti more readily resolvable. Images are max projection of 15 AT sections. More importantly, computationally calculated 3D centers of mass are more accurate and better represent the number of puncta visible after deconvolution. Scale Bar = 1 um.

Although the simulations approximate real imaged objects in a noise free environment, a real world demonstration of improved resolvability is critical. Thus, we imaged in AT a volume of microtubules, and after deconvolution (Figure 4A–C) we demonstrated that indeed the resolvability of nearby microtubules, including those that are separated by a single pixel (Figure 4C) is improved. Furthermore, the most important aspect of this work is that, because array tomography generates large and information-rich datasets, we need methods of image processing and segmentation that are simple, fast and computationally efficient. Two-dimensional Bayesian based deconvolution significantly improves the performance and accuracy of finding the weighted centers of Synapsin puncta, an abundant presynaptic protein [2], by a simple 26 neighborhood connected component analysis, in 3D volumes of cortical tissue. (Figure 4D).

### Comparison of ATD to Other Super Resolution Techniques

**Figure 5:**
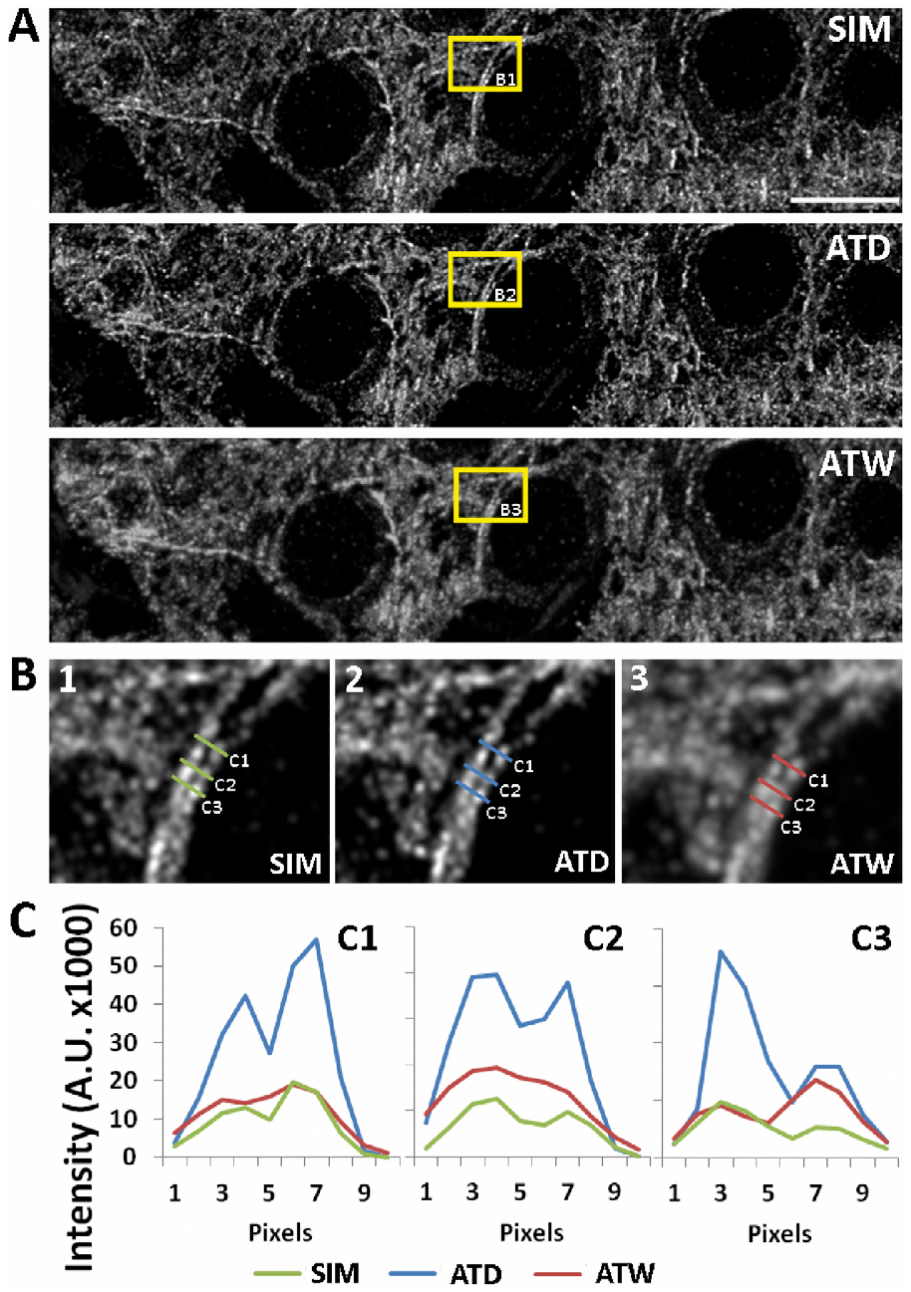
Comparison of microtubules imaged using SIM versus deconvolution. (A) Max projection of identical tissue volumes (ten 70 nm sections) imaged in SIM, ATW and ATD. Scale Bar = 10 um. (B–C) We quantify two parallel microtubules at the cross sections marked in the blow-up images in (B). Intensity cross sections (C) along the length of the microtubules show that the peaks of the microtubules are clearly resolved in the deconvolved and SIM scenario, whereas the peaks in the wide-field images are barely separated. The intensity of deconvolved image is higher, because it represents the computationally returned light at that pixel from the more blurred original image. For a more quantitative measurement of the similarity between SIM, ATD and ATW images, the Pearson's correlation coefficient is calculated using the images, which is: SIM to ATW = 0.8394. SIM to ATD = 0.8862, and ATW to ATD = 0.8982. The numbers confirm that in reference to the SIM, ATD is a closer match than ATW.

The apparent improvement of object separation in ATD images requires us to verify this result with imaging of AT ribbons using previously described and commercially available forms of super resolution microscopy. We first compared ATD with Structured Illumination Microscopy (SIM). SIM images the specimen using gratings of several orientations, which creates moiré fringes along the boundaries of the gratings. These moiré fringes provide extra spatial frequency information that can be extracted in Fourier space and used to reconstruct a new image with 100 nm resolution [18], [19]. We imaged AT ribbon arrays stained and labeled for tubulin, first using a commercial SIM, then using our wide-field AT setup. The result is a direct comparison of SIM, ATW, and ATD images of the exact same tissue volume with the exact same labeling (Figure 5). Qualitatively, the ATD images and the SIM images are virtually identical, whereas the wide-field AT image appears to have significantly lower contrast and definition (Figure 5A). Furthermore, looking at the intensity profiles of two microtubules running side by side it is clear that SIM and ATD provide similar quantitative separation of the two intensity profiles as well as matching intensity peaks and valleys, which suggest similar localization accuracy (Figure 5B–C). Finally, it is informative to look at the FT of the image volumes in the three modalities, which show that in the ATD and SIM case there is a significant increase in high spatial frequency information as demonstrated by the expansion of the magnitudes in the frequency domain (Figure S2).

**Figure 6:**
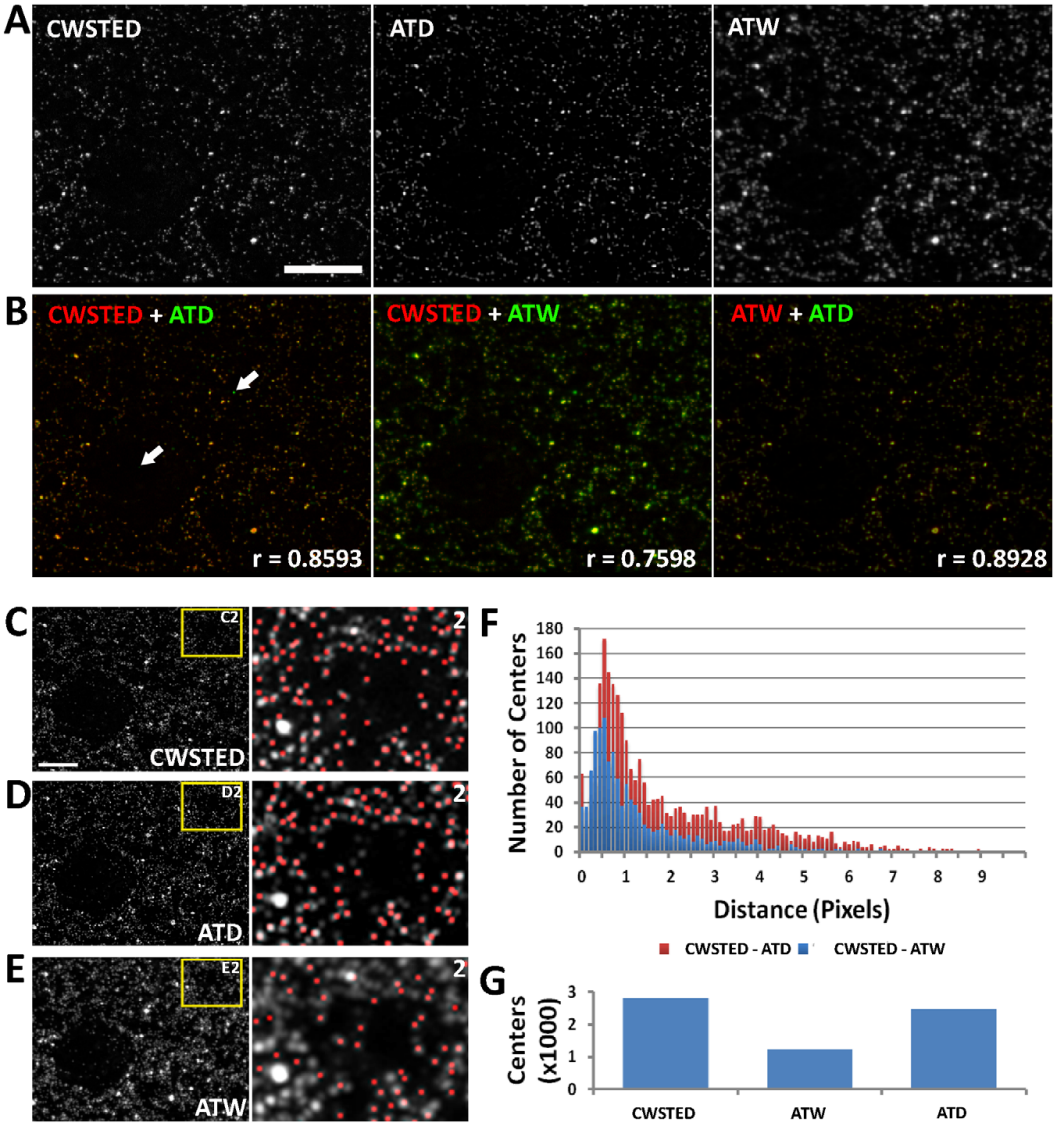
Comparison of Synapsin puncta imaged using CWSTED versus deconvolution. (A) Image of a single thin section imaged in CWSTED, ATW and ATD image. (B) Overlay of CWSTED (red) to ATW (green), CWSTED (red) to ATD (green) and ATW (red) to ATD (green). Note the high correspondence between the images, especially that of the CWSTED and ATD. The arrows in B point to apparent points in the ATD image (green) that is not in the CWSTED image (red). These few discrepancies are most likely due to the loss of primary antibody in a viscous mounting media overnight, because the CWSTED images were taken the day after the AT images, mostly because of the tendency of the CWSTED to bleach the sample during imaging. R value is the calculated Pearson's correlation coefficient between the two images. (C–E) Max projection of identical tissue volumes (ten 70 nm sections) imaged in CWSTED, ATW and ATD. Note that the CWSTED images were acquired using a 100× objective with 50 nm pixels whereas the AT images were acquired using a 63× objective with 100 nm pixels, thus the images were scaled and aligned to maintain the correct aspect ratios. Scale Bar = 5 um. (F) A distance histogram of centers of mass calculated from the CWSTED volume as compared to the ATW volume or the ATD volume. (G) A bar graph representing the total number of object centers found in the CWSTED volume, the ATW volume and the ATD volume.

Next we compared ATD to Continuous Wave Stimulated Emission Depletion microscopy (CWSTED) [20], which uses an excitation beam that is perfectly aligned with an annular depletion beam that limits the fluorescent release of photons to only a small nanometer size spot in the imaged specimen [21], [22], [20]. For this experiment, we were able to achieve 90 nm resolution with CWSTED. We imaged ribbon arrays in CWSTED and AT in a setup similar to the SIM experiments with the exception that instead of tubulin we stained the brain tissue for Synapsin. Again, the CWSTED and ATD images are extremely similar by visual comparison (Figure 6A, B). More importantly, the locations of the calculated centers of mass using CWSTED and ATD are similar, even with the expected jitter caused by the alignment and scaling of images due to the differences in the two imaging setups (100× objective with 50 nm pixels for CWSTED and 63× objective with 100 nm pixels for AT) (Figure 6C–E). A histogram of point to point distances between the modalities shows that the majority of points are within 1.5 pixels of each other (Figure 6F). The most striking difference between ATW and ATD in comparison to CWSTED is the number of objects computationally segmented in the image volume using 3D connected component analysis, with ATW lagging CWSTED and ATD due to the poor 3D object separation in the image volume (Figure 6G).

**Figure 7:**
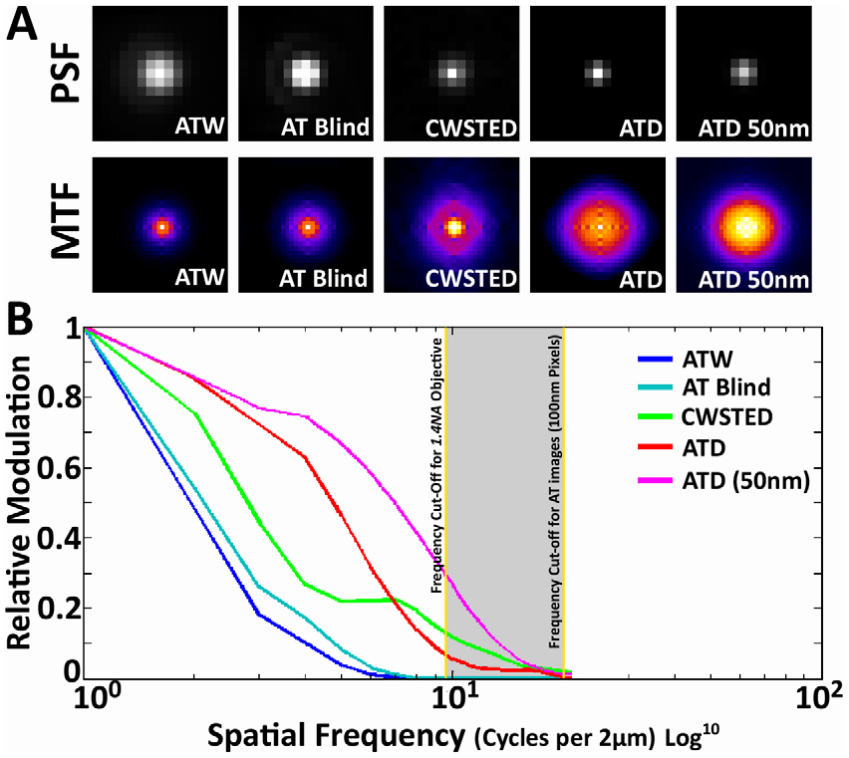
Comparison of measured MTFs. (A) Top row: the PSFs of ATW, AT with blind deconvolution, STED and ATD. Bottom row: the calculated MTF of the above modalities, which we accomplished by performing a FT on the PSF and graphing the modulus or the real component of the FT as a 2D intensity plot. (B) A plot of the rotationally averaged MTFs of ATW, AT with blind deconvolution, ATD and CWSTED. Yellow lines denote the theoretical cut-off frequency for a 1.4NA objective is calculated using 2NA/λ (λ = wavelength-488 nm, NA = numerical aperture-1.4). The grey region represents the frequency domain that is between the diffraction limit and the pixel limit of AT images. The frequencies in this region are not present in the actual recorded image, and ATD's MTF extension into this region must be accounted for purely through analytical continuation by RL. In contrast, blind deconvolution does not apply analytical continuation and remains bandwidth limited by the cut-off frequency. CWSTED on the other hand clearly surpasses the diffraction limit and has 50 nm pixels. Further tests using ATD with 50 nm pixels (63× objective with 1.6× optivar) reveal that the MTF extension can be further pushed by allowing analytical continuation to continue further using a smaller pixel size.

**Figure 8:**
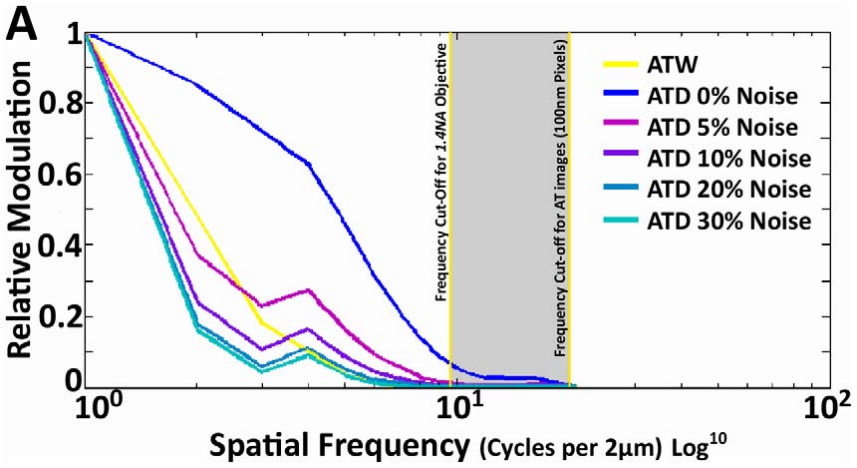
The effect of noise on the spatial frequency recovery of RL deconvolution. (A) This graph clearly illustrates the effect of Poisson noise on the fidelity of RL deconvolution. Poisson noise was artificially generated and added to the same image stack, and the RL was performed on those images, then the MTF of the images were calculated to demonstrate that even with a 5% injection of noise the spatial frequency recovery was significantly degraded, and for a 10% noise increase for most frequencies of the MTF the ATD is not better than ATW. Pixel size of the images were 100 nm.

Finally, it is of interest to look at the empirical OTFs of the above modalities. More specifically, we are interested in the modulus of the OTF or the Modulation Transfer Function (MTF), which describes the amount of signal power present at each spatial frequency, or more practically, the amount of contrast that can be generated for each spatial frequency and relates directly to the resolvability of that spatial frequency in the actual image. The measured MTF was generated by applying FT to PSFs generated with 100 nm beads imaged at 488 nm wavelength for AT images and single sub-diffraction primary with secondary fluorescent antibodies at 488 nm in CWSTED. The MTF of ATW falls off dramatically as we approach the theoretical cut-off frequency of a 1.4NA objective (Figure 7). The cut-off frequency is described by the equation 2NA/λ (λ = wavelength, NA = numerical aperture). This clearly demonstrates the bandwidth-limited nature of the MTF in AT imaging. Two dimensional blind deconvolution of the ATW images increases the amount of signal at the higher spatial frequencies, but it only serves to bring the MTF edge closer to the theoretical cut-off (Figure 7). CWSTED's major gain in the MTF is at the higher spatial frequencies and as expected for a super resolution technique it surpasses the cut off value (Figure 7). The most significant aspect of the ATD MTF is the dramatic increase in modulation at all frequencies within the frequency cut-off. This massive improvement in modulation is the most likely cause of the image improvement seen in ATD, however intriguingly the ATD MTF, like CWSTED was able to extend beyond the frequency cut-off of the objective. The MTF of the actual AT images are bandwidth limited by diffraction, but it appears that in ATD, our deconvolution algorithm has mathematically extended the high spatial frequency information, which does eventually hit a hard limit, that is set by the image pixel size (100 nm), whereas CWSTED does not (pixel = 50 nm) (Figure 7). Further testing of ATD with 50 nm pixels using a 1.6× optivar and the 63× objective revealed that the higher spatial frequency component can be further pushed out approaching CWSTED levels (Figure 7). While this is a curious result and has interesting implications to the interpretation of our result, this phenomenon has been demonstrated in astronomical imaging. RL, but not blind deconvolution, applied to images with high signal to noise and band-limited OTFs can recover, through analytic continuation in the Fourier domain, frequency information beyond that of the measured object, thus allowing the extension of the MTF beyond the diffraction limit [23]–[25], [15]. Analytic continuation is a method in complex analysis that allows the extension of the domain over which a function is defined [26], [23], [25], [27]. Analytic continuation requires an original function to be analytic within its domain of definition, and not every complex function is analytic. In essence analytic continuation states that knowing the value of a complex function in some finite complex domain uniquely determines the value of the function at every other point. In image restoration, if a 2D object is compact in the space domain, i.e., confined within a finite region, its FT is analytic [28], [29]. In wide-field fluorescence images with diffraction limited OTFs, the image is an analytic function restricted to the pass-band, which analytic continuation maybe be applied to extrapolate it beyond the pass-band [23], [25], [30]. In practice, analytic continuation is highly sensitive to noise [26], [23], [31], [32] (Figure 8), and applied without constraints on real images results in little resolution improvement [33]. However, if we apply the reasonable constraint that all observations in our images are non-negative, which is an intrinsic assumption in RL, significant improvements in resolution can be obtained even with a moderate signal to noise ratio [23], [16], [25].

**Figure 9:**
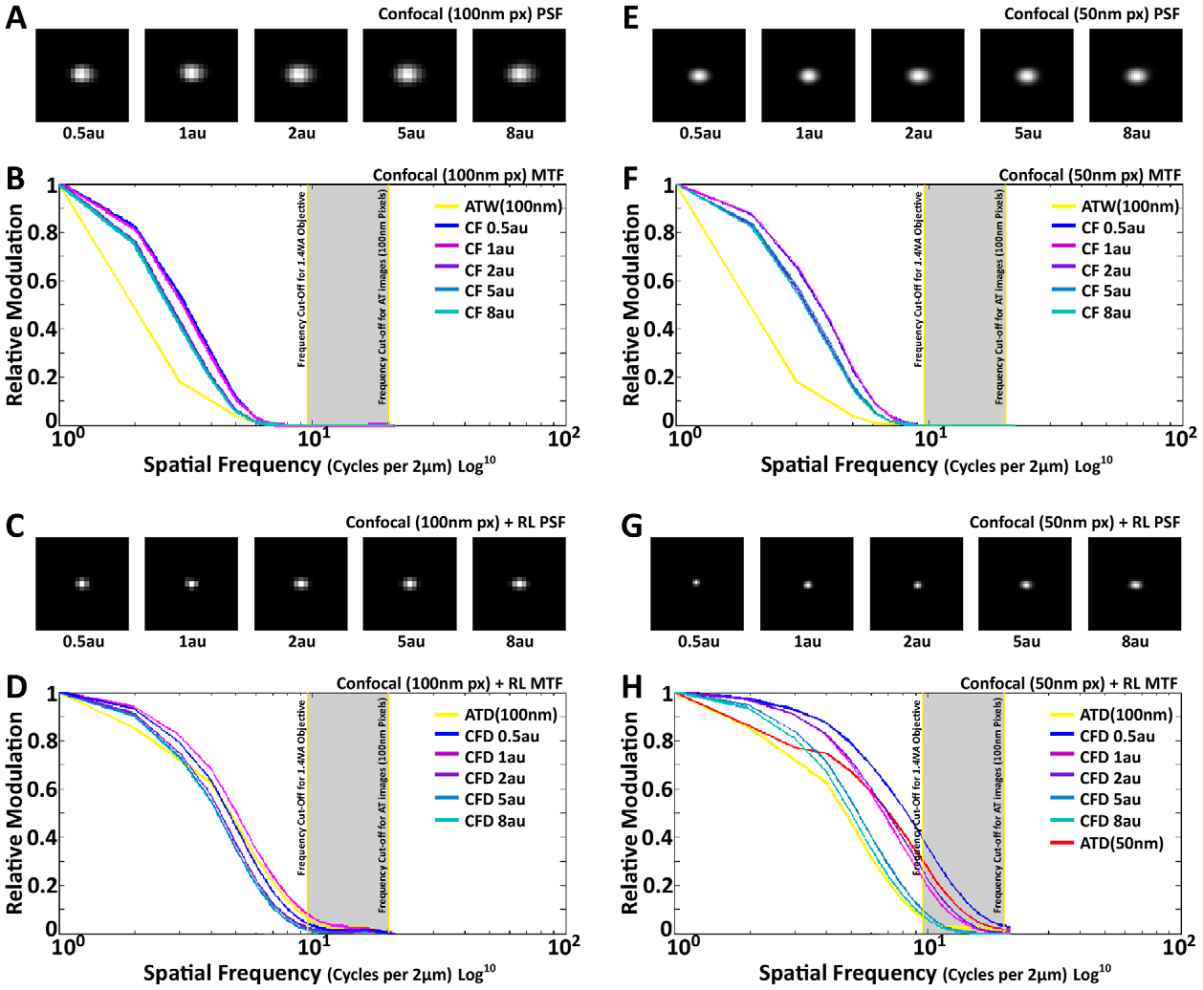
RL deconvolution of confocal images. (A, C, E, G) Images of Confocal (CF) PSFs at different pinhole sizes for different pixels sizes and after deconvolution. Each PSF is an average of ∼200 individual images from 110 nm fluorescent beads. (B) MTF plot of CF with 100 nm pixels at different pinhole sizes, compared to ATW with 100 nm pixels. Note the increase in MTF magnitude for all CF cases as compared to ATW. (D) MTF of RL deconvolved confocal images (CFD) at 100 nm pixels as compared to ATD with 100 nm pixels. The magnitude of the MTF is comparable at the high spatial frequencies with CFD performing better at lower spatial frequencies. (F) MTF plot of CF with 50 nm pixels, which is very similar to (B), with a slight increase in MTF frequency. (H) MTF plot of CFD at 50 nm pixels, as compared to ATD at 100 nm and 50 nm pixels. It is clear that CFD at 50 nm completely out performs ATD at 100 nm. ATD at 50 nm perform closely with CFD in the high spatial frequencies, with the exception of CFD at 0.5 airy unit (au). At lower spatial frequencies, as seen in (D) as well, CFD out performs ATD.

Finally we thought it might be of interest to test whether deconvolution of confocal images from our thin sections would improve our results further, because the confocal PSF is the multiplication of the excitation PSF and the emission PSF, which sharpens the lateral PSF and improves lateral resolution. Empirically we show, as we stated earlier, confocal have better native lateral resolution and spatial frequency capture, as can be seen in its MTFs as compared to ATW (Figure 9 A, B, E, F). Moreover, as one expects, by decreasing the pinhole size the MTF does see an appreciable increase in all spatial frequencies (Figure 9 A, B, E, F). RL deconvolution of confocal images, much like ATD, allowed the extension of spatial frequencies beyond the cut-off limit of the objective, especially when 50 nm pixels were used, and in some cases (when the pinhole is 1 airy unit (au) or smaller), RL plus confocal actually out performs ATD (Figure 9 D, H). This suggests that for array tomography, confocal imaging is a viable alternative to wide-field, although the gain in spatial frequency capture and recovery might not outweigh the increased image acqusition time, equipment cost and illumination intensity (especially, for small pinhole sizes (< = 1au) where confocal deconvolution beats ATD).

## Discussion

It must be noted that although our comparison of ATD with commercial SIM and CWSTED appear to suggest that ATD images in certain instances can approach the resolution of those techniques, we must caution that ATD is purely a mathematical process based on reasonable, but not perfect assumptions. It does not record extra spatial frequencies as SIM and CWSTED does through the use of deterministic light patterns. Moreover, the proper implementation of RL requires that the algorithm to converge through the iterations [34], and although in practice applying RL to AT images has always converged, one must be aware that this is a mathematical process that can fail, and the results of any deconvolution must be carefully interpreted. That said, the ideal optical characteristics of ultra-thin (50–100 nm) sectioning (minimal non-linear aberrations, optimal refractive index correction, ideal flatness of field, high signal to noise and a spatially invariant PSF) creates optimal circumstances for two-dimensional Bayesian based deconvolution (RL) to dramatically improve the MTF of AT images and perhaps even mathematically extend it, thus improving the resolution and computational segmentation of imaged protein structures. Our application of deconvolution, in the AT framework, truly allows RL to shine, because of the ideal data characteristics, which in many ways mimic the astronomical images that RL was originally designed for. Interestingly this does suggest that optical methods, such as evanescent field microscopy, that have extremely fine optical sectioning, could also benefit greatly from RL deconvolution.

The combination of deconvolution and AT creates volumetric images of intact tissue with a combination of speed, resolution, coverage and cost that cannot be matched by any other imaging modality. This coupled with the highly multiplexed imaging of proteins that is native to the AT procedure opens the door for the detection of biologically relevant protein localization in intact tissue samples at a scale and detail that will be crucial for understanding the function and dysfunction of biological systems. This spatial proteomic approach, where protein localization is maintained with sub-organelle precision from the *in-vivo* context can provide an essential piece of information that is missing in traditional proteomic approaches. It has become increasingly clear that the analysis of total expression level of proteins lacks the nuance that will be required to understand function at a complex cellular and systems level. The localization of a protein within a cell in relation to other proteins within its interaction repertoire is as important to the function of that protein as its modification state or its intrinsic structural and catalytic capabilities. The collection and analysis of this data is the information space that is uniquely occupied by ATD. It is this convergence of proteomic breadth with sub-organelle localization accuracy that will allow a much deeper analysis of biological function that can contribute significantly to our understanding of biological processes.

## Methods

### Preparation of AT Ribbon Arrays

Tissue preparation, array creation and immunohistochemistry are described in detail in previous publications [1], [2]. In short, a small piece of tissue (∼2 mm high by 1 mm wide by 1 mm deep), in our case cortical tissue from the somatosensory cortex of the mouse brain, is microwave fixed in 4% Paraformaldehyde. The fixed tissue is then dehydrated in graded steps of ethanol, and then embedded in LR White resin overnight at 50°C. The embedded tissue is section on an ultramicrotome at a thickness of 70 nm and placed as a ribbon array directly on gelatin or carbon coated glass coverslips.

Immunohistochemistry is then carried out on the arrays using primary antibodies targeting antigens of choice (alpha-Tubulin, *Abcam* ab18251 and Synapsin, *Cell Signaling Technology* 5297S). The primary antibodies are visualized via fluorescently labeled secondary antibodies (Alexa 594, *Invitrogen* A11037, Alexa 488, *Invitrogen* A11034, and Alexa 647, *Invitrogen* A21245), and mounted in SlowFade Gold antifade with DAPI (*Invitrogen*).

### Microscopy

Wide-field imaging of ribbons were accomplished on a *Zeiss* Axio Imager.Z1 Upright Fluorescence Microscope with motorized stage and Axiocam HR Digital Camera as previously described [1], [2]. A position list was generated for each ribbon array of ultrathin sections using custom software modules written for Axiovision. Single fields of view were imaged for each position in the position list using a *Zeiss* 63×/1.4 NA Plan Apochromat objective.

SIM imaging of ribbons were performed on a *Zeiss* ELYRA PS.1 super resolution scope using an *Andor* iXon 885 EMCCD camera. Positions on the ribbons were manually acquired across each section of the ribbon, and each fluorescent channel was imaged with five pattern rotations with 5 translational shifts, using a *Zeiss* 63×/1.46 NA Plan Apochromat objective. The final SIM image was created using modules build into the Zen software suite that accompanies the imaging setup.

CWSTED imaging was performed on a *Leica* TCS SP5 II using *Lecia* HyD hybrid PMT detectors. Positions on the ribbons were manually acquired across each section of the ribbon, and CWSTED images were acquired with a calibrated 90 nm resolution using a *Lecia* HCX PL APO 100× 1.40NA objective.

Confocal imaging was performed on a *Zeiss* LSM-510 using a *Zeiss* 63×/1.46 NA Plan Apochromat objective. Images of 100 nm beads, seeded on AT thin sections, were acquired using manually set pin-hole sizes ranging from 0.5 airy unit to 8 airy unit using either 100 nm pixels or 50 nm pixels.

### Image Registration and Processing

Image stacks from ATW, SIM and STED were imported into *FIJI* and aligned using both rigid and affine transformations with the Register Virtual Stacks plugin. The aligned image stacks were further registered across image sessions using MultiStackReg.

The aligned and registered image stacks were imported into Matlab (*Mathworks*) and deconvolved using the native implementation of Richardson-Lucy deconvolution with empirical or theoretical PSFs with 10 iterations [15]. Custom functions were written to automate and facility this work flow. Blind deconvolution is also natively implemented in Matlab.

Matlab native function (regionprops) was used to calculate the centers of mass of punctas in the image volumes using 26 neighborhood 3D connected component analyses with an assumed background threshold that is 0.1 of the total dynamic range, which is 6553.5 for a 16bit image, and is in line with previous background thresholds used for AT analysis [2]. Custom functions were implemented to facility the handling and processing of the data.

## Supporting Information

Figure S1 Deconvolution improves the resolvability of adjacent non-similar point sources. Here we plot the central cross-sectional profiles of two empirically measured point source to simulate adjoining point sources one that is half the intensity of the other. Through the linear addition of intensity profiles after the two point sources were shifted in space, we demonstrate the improved resolution of the point sources after deconvolution. Note: 0 along the x axis denotes the center of the two point sources. (A) The centers are shifted by 1 pixel (100 nm) apart, and predictability one cannot resolve the two points in either case, because the centers occupy adjacent pixels. (B) The centers are shifted by 2 pixels (200 nm) apart, and now clearly the deconvolved point sources are resolvable. (C) The centers are shifted by 3 pixels (300 nm) apart. The situation is not different from the 2pixel shift. (D) The centers are shifted by 4 pixels (400 nm), and finally, the non-deconvolved point sources are resolvable. Thus, deconvolution decreases the threshold of resolvability for 4 pixel shift to 2 pixel shift.
(TIF)

Figure S2 Fourier Transforms of ATW, ATD and SIM Image Volumes. (A–C) Representative max projection images of ATW, ATD and SIM image volumes (ten 70 nm sections: above) presented with the Fourier Transforms (FT) of the Image Volumes as performed in FIJI (below). The false colored FT images represent the spatial frequency information present in the image, and the magnitude of the frequency component in the image is represented by intensity in the image. The center of the image is the mean frequency component of the image, and as we move further from the center of the image the intensities represent the magnitude of higher and higher spatial frequencies present in the image. Note the large increase in high spatial frequency information in the SIM and ATD images as compared to the ATW images. Scale Bar = 5 um.
(TIF)
