# Supplementary figures and images for: Sub-diffraction Limit Localization of Proteins in Volumetric Space Using Bayesian Restoration of Fluorescence Images from Ultrathin Specimens
Source: PLoS Comput Biol. 2012 Aug 30;8(8):e1002671. doi: 10.1371/journal.pcbi.1002671 (PMC3431294; doi:10.1371/journal.pcbi.1002671)

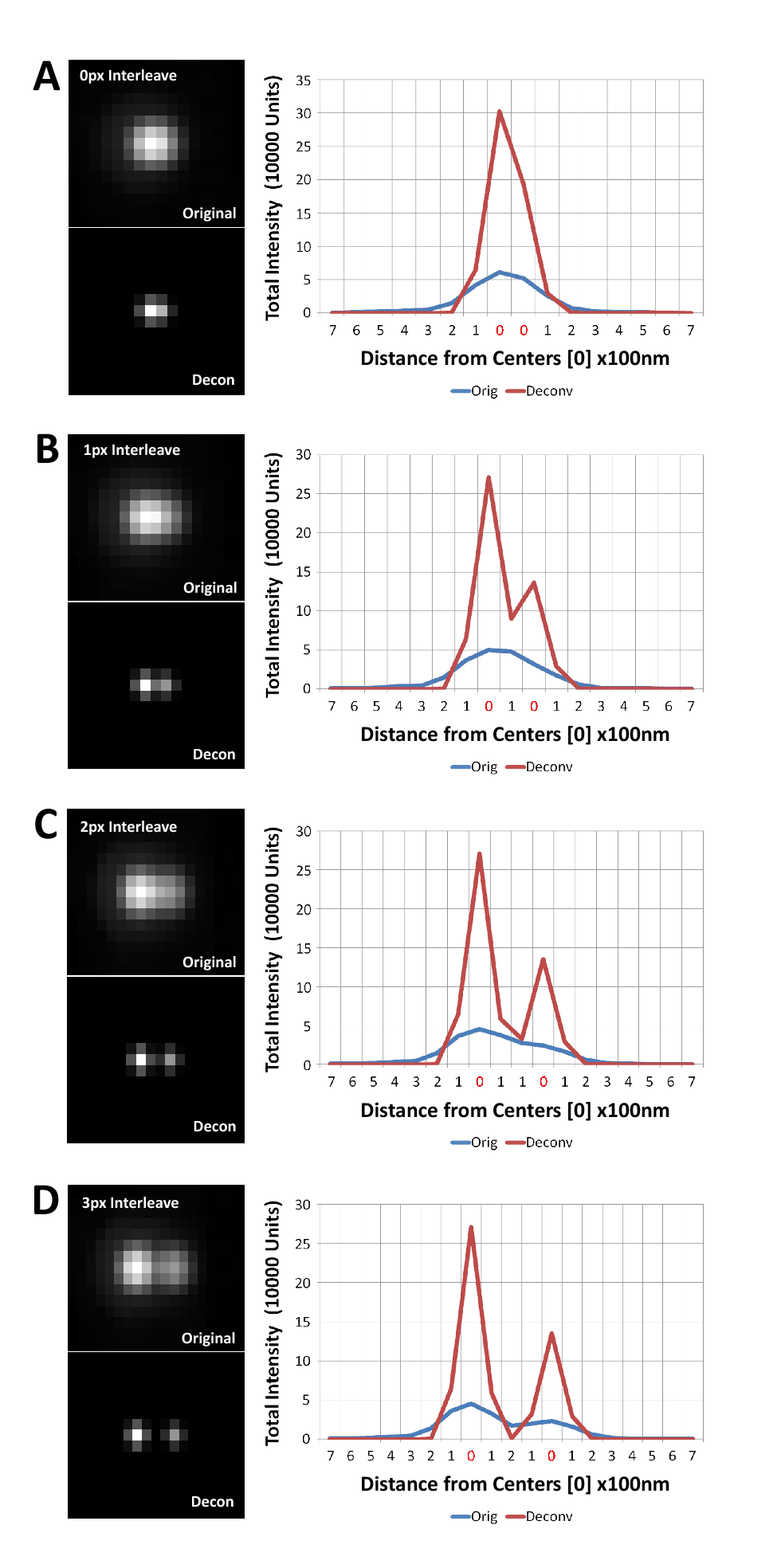

Supplement: Figure S1 — Deconvolution improves the resolvability of adjacent non-similar point sources. Here we plot the central cross-sectional profiles of two empirically measured point source to simulate adjoining point sources one that is half the intensity of the other. Through the linear addition of intensity profiles after the two point sources were shifted in space, we demonstrate the improved resolution of the point sources after deconvolution. Note: 0 along the x axis denotes the center of the two point sources. (A) The centers are shifted by 1 pixel (100 nm) apart, and predictability one cannot resolve the two points in either case, because the centers occupy adjacent pixels. (B) The centers are shifted by 2 pixels (200 nm) apart, and now clearly the deconvolved point sources are resolvable. (C) The centers are shifted by 3 pixels (300 nm) apart. The situation is not different from the 2pixel shift. (D) The centers are shifted by 4 pixels (400 nm), and finally, the non-deconvolved point sources are resolvable. Thus, deconvolution decreases the threshold of resolvability for 4 pixel shift to 2 pixel shift. (TIF) [file pcbi.1002671.s001.tif]

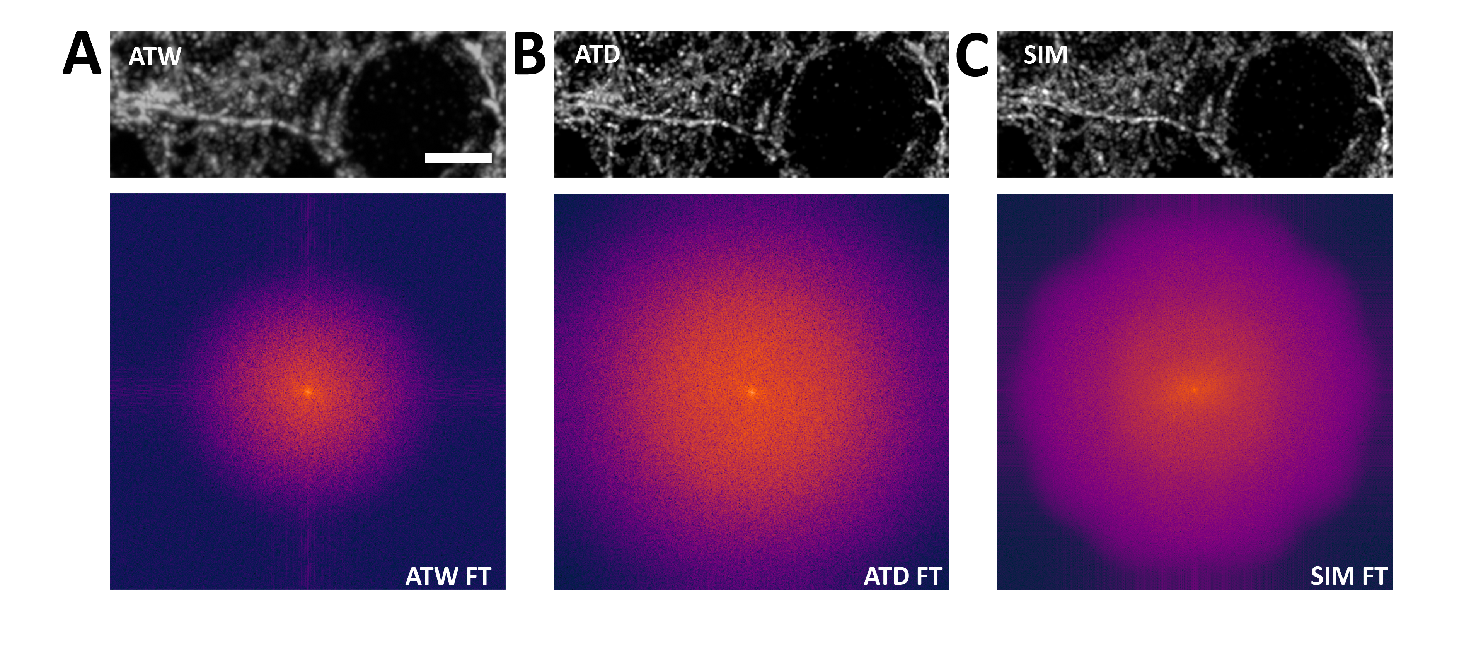

Supplement: Figure S2 — Fourier Transforms of ATW, ATD and SIM Image Volumes. (A–C) Representative max projection images of ATW, ATD and SIM image volumes (ten 70 nm sections: above) presented with the Fourier Transforms (FT) of the Image Volumes as performed in FIJI (below). The false colored FT images represent the spatial frequency information present in the image, and the magnitude of the frequency component in the image is represented by intensity in the image. The center of the image is the mean frequency component of the image, and as we move further from the center of the image the intensities represent the magnitude of higher and higher spatial frequencies present in the image. Note the large increase in high spatial frequency information in the SIM and ATD images as compared to the ATW images. Scale Bar = 5 um. (TIF) [file pcbi.1002671.s002.tif]
